# Wnt5a enhances proliferation of chronic lymphocytic leukemia and ERK1/2 phosphorylation via a ROR1/DOCK2-dependent mechanism

**DOI:** 10.1038/s41375-020-01055-7

**Published:** 2020-10-23

**Authors:** Md Kamrul Hasan, Emanuela M. Ghia, Laura Z. Rassenti, George F. Widhopf, Thomas J. Kipps

**Affiliations:** Center for Novel Therapeutics, Moores Cancer Center, University of California San Diego, La Jolla, CA USA

**Keywords:** Chronic lymphocytic leukaemia, Cell signalling

## Abstract

Patients with chronic lymphocytic leukemia (CLL) have high plasma-levels of Wnt5a, which can induce phosphorylation of ERK1/2 and enhance CLL-cell proliferation. Such effects could be inhibited by treatment with an ERK1/2 inhibitor, ERK1/2-specific siRNA, or cirmtuzumab, an anti-ROR1 mAb. The CLL-derived line, MEC1, expresses Wnt5a, but not ROR1. MEC1 cells transfected to express ROR1 (MEC1-ROR1) had higher levels of phosphorylated ERK1/2 than parental MEC1, or MEC1 transfected with ROR1ΔPRD, a truncated ROR1 lacking the cytoplasmic proline-rich domain (PRD), or ROR1^P808A^ a mutant ROR1 with a P→A substitution at 808, which is required for complexing with the Rac-specific-guanine-nucleotide-exchange factor DOCK2 upon stimulation with Wnt5a. We silenced DOCK2 with siRNA and found this repressed the capacity of Wnt5a to induce ERK1/2 phosphorylation in MEC1-ROR1 or CLL cells. CLL cells that expressed ROR1 had higher levels of phosphorylated ERK1/2 or DOCK2 than CLL cells lacking ROR1. Although we found ibrutinib could inhibit the phosphorylation of ERK1/2 and DOCK2 induced by B-cell-receptor ligation, we found that this drug was unable to inhibit Wnt5a-induced, ROR1-dependent phosphorylation of ERK1/2 or DOCK2. This study demonstrates that Wnt5a can induce activation of ERK1/2 and enhance CLL-cell proliferation via a ROR1/DOCK2-dependent pathway independent of BTK.

## Introduction

Chronic lymphocytic leukemia (CLL) is characterized by the accumulation of monoclonal B cells in the marrow and lymphoid tissues, where leukemia cells receive growth and survival signals within the tumor microenvironment [1, 2]. Factoring in such signaling is the B-cell receptor (BCR), which is comprised of a ligand-binding transmembrane immunoglobulin molecule and accessory proteins CD79a and CD79b. Ligation of the BCR induces activation of kinases, such as Bruton’s tyrosine kinase (BTK), leading to downstream activation of extracellular signal-regulated kinase (ERK)1/2, which can promote CLL-cell survival and proliferation [3–5]. Similarly, chemokines, such as CXCL12, can induce activation of ERK1/2 via stimulation of chemokine-receptors, such as CXCR4, that also are dependent on BTK [6, 7]. The importance of ERK1/2 activation by the microenvironment is underscored by studies showing that inhibitors of MEK1/2, such as MEKi-1, which block ERK1/2 activation, can abrogate the survival signaling provided to leukemia cells by the microenvironment [8]. Moreover, the importance of BCR- and chemokine-receptor signaling is highlighted by the clinical activity of drugs that inhibit BTK, such as ibrutinib [2].

Other signaling pathways also can activate ERK1/2 in CLL cells independent of BCR- or chemokine-receptor signaling. For example, ligation of CD5 can induce phosphorylation of ERK1/2 via a pathway that appears dependent on the entry of extracellular calcium (Ca^2+^), which is facilitated by upregulation of the transient receptor potential channel 1 (TRPC1) [9]. Furthermore, the non-canonical Wnt factor, Wnt5a, which can be found at high levels in the plasma of patients with CLL [10, 11], can induce activation of ERK1/2 in neoplastic cells [12]. This is notable as Wnt5a is an identified ligand for ROR1 [13], an evolutionarily conserved and developmentally-restricted type-I surface protein, which is expressed by the leukemia cells of most patients with CLL [13–15]. Moreover, ROR1 signaling induced by Wnt5a can promote CLL-cell survival, growth, and migration, which can be inhibited by cirmtuzumab, a mAb specific for the extracellular domain of ROR1 [10, 16]. Conceivably, Wnt5a can induce ERK1/2 activation in CLL cells via a ROR1-dependent pathway. Consistent with this notion is the recent study in a related ROR1-expressing hematologic malignancy, namely mantle cell lymphoma (MCL), that showed that silencing ROR1 could repress activation of ERK1/2 in MCL cells [17]. Therefore, we investigated whether Wnt5a could activate ERK1/2 and whether such activation was dependent on expression of ROR1.

## Materials and methods

### Immunoblot analysis

Immunoblot analysis was performed as described [18, 19]. Equal amounts of total protein from each sample were separated by SDS-PAGE and blotted onto polyvinylidene difluoride membrane. Immunoblot analysis was performed using primary mAbs specific for ROR1 (Cell Signaling Technology, Danvers, MA, USA, Cat# 4102), DOCK2 (Santa Cruz, Dallas, TX, USA; Cat# sc365242), Phosphotyrosine (4G10, Millipore-Sigma, Burlington, MA, USA; Cat# 05–321), ERK1/2 (Cell Signaling; Cat# 9102), pERK1/2 (Thr202/Tyr204) (Cell Signaling; Cat# 9101), AKT (Cell Signaling; Cat# 9272), pAKT (Ser473) (Cell Signaling; Cat# 9271), or β-Actin (Cell Signaling; Cat# 4967), which were detected using secondary antibodies conjugated with horseradish peroxidase (Cell Signaling Technology).

### RNA-sequencing and gene set enrichment analysis

PBMC of 3 CLL samples were collected before therapy (Pre-Rx) and at day 28 of cirmtuzumab treatment (D28). Each D28 sample was collected after patients had received 2 doses of 16 mg/kg cirmtuzumab. Negative isolation of CLL cells to ≥95% purity was performed prior to RNA isolation. Total RNA was extracted using TRIzol reagent (Life Technologies). Data were analyzed as previously described [11]. Gene set enrichment analysis (GSEA) compared pre-treatment (Pre-Rx) and day 28 (D28) levels for the same patients in the 16 mg/kg cohort (*n* = 3). Using GSEA software [20], we conducted GSEA on the primary RNA-seq data pre-Rx and day 28 of cirmtuzumab treatment. We focused GSEA on genes regulated by transcription factors activated by phosphorylated ERK1/2 [21], namely genes targeted by E-twenty-six (Ets-1) [21, 22], E-26-oncogene (Elk) [21], Hypoxia-inducible factor-1 (HIF-1) [23], Jun [24], CREB [25], or Fos [21]. Each gene set was considered significant when the false discovery rate (FDR) was <25% [20]. The FDR *q* value was adjusted for gene set size and multiple hypothesis testing.

### Study approval

CLL cells were collected at the Moores Cancer Center from patients who provided written, informed consent, and who satisfied diagnostic and immuno-phenotypic criteria for CLL. This study was performed in compliance with the Declaration of Helsinki and approval of the UCSD Institutional Review Board (IRB) (IRB approval number 080918).

## Results

### Wnt5a induces ROR1-signaling to activate ERK1/2 and promote CLL-cell proliferation

**Fig. 1:**
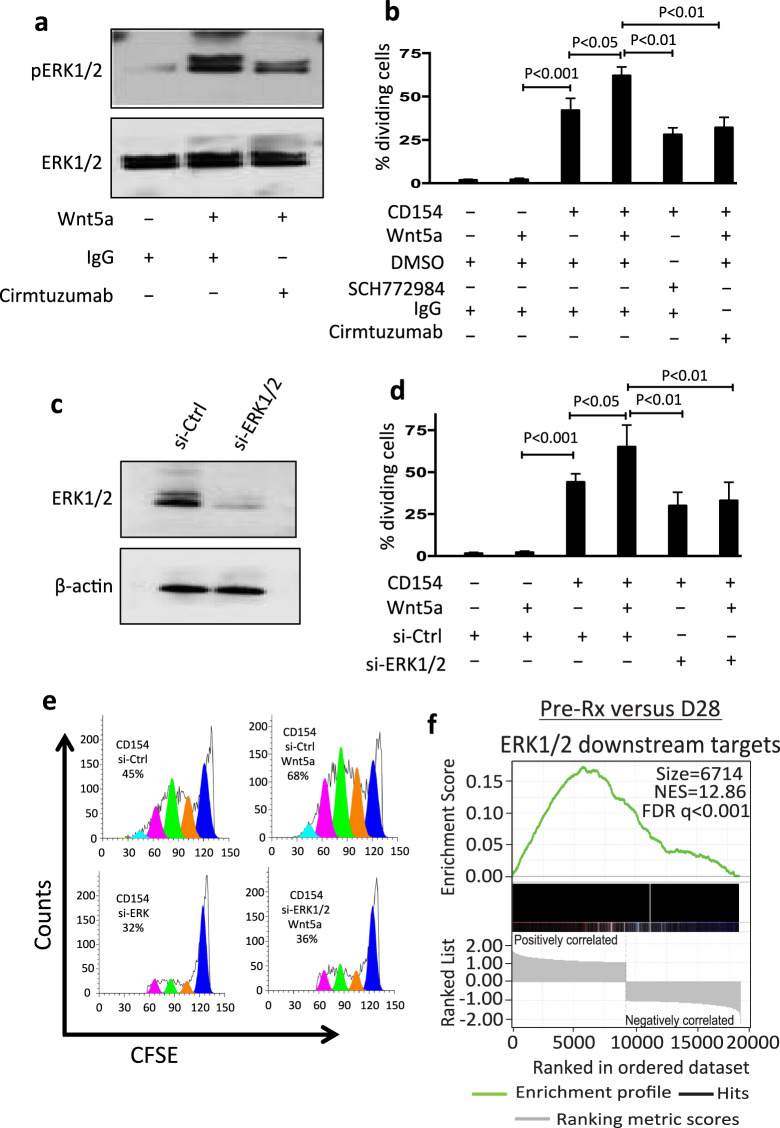
ERK1/2 factors in the capacity of Wnt5a to enhance CLL proliferation. **a** Immunoblot analysis of lysates prepared from primary CLL cells (representative of three patients) pretreated without (-) or with (+) cirmtuzumab (20 μg/ml) for 2 h and stimulated without (-) or with (+) Wnt5a (100 ng/ml) for 5 min; expression of total ERK1/2, and activated pERK1/2 was measured, as indicated on the left. **b** Mean proportions of dividing CLL cells from each of 6 patients (3 U-CLL and 3 M-CLL) under conditions indicated at the bottom. *P* < 0.05; *P* < 0.01; *P* < 0.001, as assessed by 2-tailed Student’s *t* test. **c** CLL cells transfected with control siRNA or siRNA targeting ERK1/2 and examined 72 h later by western-blot (representative of 3 patients). Cell viability was ≥90% before siRNA transfection and ≥80% in either control or ERK1/2-siRNA transfected cells. **d** Mean proportions of dividing CLL cells from each of 6 patients (3 U-CLL and 3 M-CLL) under conditions indicated at the bottom. *P* < 0.05; *P* < 0.01; *P* < 0.001, as assessed by 2-tailed Student’s *t* test. **e** Fluorescence of CLL cells stained with CFSE and treated with CD154, without (–) or with (+) Wnt5a. The percentage of dividing cells is indicated in each histogram. **f** Gene set enrichment analysis (GSEA) on the transcriptomes of paired pre-treatment (Pre-Rx) and post-treatment CLL cells at D28 (D28) evaluating for pre and post-treatment differences in the expression of ERK1/2 downstream target genes. Gene set size (SIZE), normalized enrichment score (NES), and FDR *q* value (FDR q) are indicated.

We treated serum-starved CLL cells with or without Wnt5a and assessed for activation of ERK1/2. We found that treatment with Wnt5a for 5 min induced phosphorylation of ERK1/2 (Fig. 1a). Moreover, the anti-ROR1 mAb cirmtuzumab (20 μg/ml), which specifically can block ROR1-signaling [10, 11], could inhibit the capacity of Wnt5a to induce activation of ERK1/2.

In prior studies we demonstrated that Wnt5a could enhance the proliferation of CLL cells that expressed ROR1 when added to serum-starved CLL cells co-cultured with IL-4, IL-10, and HeLa cells engineered to express CD154 [10, 16]. In this study, we found that treatment of such cultures with the ERK1/2 inhibitor, SCH772984 [26, 27], or cirmtuzumab, inhibited the capacity of Wnt5a to enhance proliferation of the CLL cells, regardless of whether they used unmutated (U) or mutated (M) immunoglobulin heavy-chain variable region genes (*IGHV*) (Fig. 1b), suggesting that activation of ERK1/2 was required for the enhanced CLL-cell proliferation and that such activation was ROR1-dependent. Consistent with this notion, we found that silencing *ERK1/2* with siERK1/2 siRNA (Fig. 1c), while not acutely affecting cell viability, also impaired the capacity of Wnt5a to enhance CLL-cell proliferation relative to that of CLL cells treated with control, nonspecific siRNA (siCtrl) (Fig. 1c-e).

Because cirmtuzumab could block Wnt5a-induced activation of ERK1/2, we examined whether treatment with cirmtuzumab could repress expression of genes induced by activated ERK1/2 in vivo. For this we used gene-set-enrichment analyses (GSEA) to examine the transcriptomes of leukemia cells collected prior to therapy and on D28 of treatment with cirmtuzumab (*n* = 3) [9]. These analyses revealed that expression levels of genes induced by activated ERK1/2 are significantly greater in the CLL cells of patients prior to therapy than in the CLL cells from the same patients after treatment with cirmtuzumab (Fig. 1f). Moreover, compared to matched pre-treatment transcriptome samples, the D28 post-treatment transcriptome samples had a significant repression of 5 of 6 gene sets targeted by transcription factors, namely genes targeted by E-twenty-six (Ets-1), E-26-oncogene (Elk), Hypoxia-inducible factor-1 (HIF-1), Jun, CREB, or Fos (Supplementary Fig. S1A-F).

### Proline 808 of ROR1 is crucial for Wnt5a-induced activation of ERK1/2

**Fig. 2:**
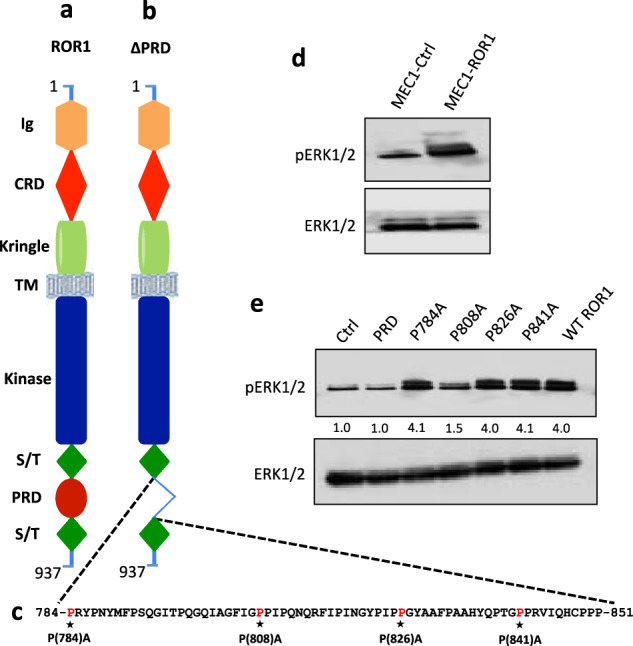
ROR1^P(808)A^ has impaired capacity to induce ERK1/2 phosphorylation. **a** Schematic depicts the structure of ROR1 protein with different domains.**b** ΔPRD represents the truncated form of ROR1 without its proline rich region. **c** Amino acid sequences of the proline rich domain of ROR1. Asterisks indicate the proline (P) amino acid residues that had been substituted with alanine (A). **d** Immunoblot analysis of lysates prepared from MEC1-Ctrl or MEC1-ROR1 cells, as indicated on the top; the membranes were probed with anti-phospho ERK1/2 or anti-ERK1/2 antibody, as indicated on the left. **e** Activation of ERK1/2 was measured by immunoblot analysis, using lysates of MEC1-Ctrl, MEC1-ΔPRD, MEC1-ROR1 (W/T) or MEC1 cells transfected with each of the various mutated forms of ROR1, as indicated on the top; the membranes were probed with anti-phospho ERK1/2 or anti-ERK1/2 antibody, as indicated on the left. The numbers between two lanes are ratios of band integrated optical density (IOD) of pERK1/2 relative to total ERK1/2, and normalized with respect to that of MEC1-Ctrl cells.

We examined for ERK1/2 activation in MEC1 cells, which were derived from CLL cells and have been used as a model system for study of CLL [28]. In prior studies we found that MEC1 does not express ROR1 [29], but does express high levels of Wnt5a, obviating use of exogenous Wnt5a [30]. Consistent with the findings that Wnt5a can induce activation of ERK1/2 via a ROR1-dependent pathway, MEC1 cells made to express ROR1 via transfection with a plasmid ROR1-expression vector had significantly higher levels of phosphorylated ERK1/2 than mock-transfected MEC1, or MEC1 cells transfected with an empty, control-plasmid vector (Fig. 2d).

We studied the levels of phosphorylated ERK1/2 in MEC1 cells transfected to express different mutant constructs of ROR1, including ROR1^ΔPRD^ lacking the cytoplasmic proline-rich-domain (PRD), or ROR1^P784A^, ROR1^P808A^, ROR1^P826A^, or ROR1^P841A^, which have point mutations resulting in proline (P) to alanine (A) substitutions at –P-X-X-P- motifs within the PRD, resulting in a P→A substitution at position 784, 808, 826, or 841, respectively (Fig. 2a-c and Supplementary Fig. S2). We found that MEC1 cells expressing ROR1^P784A^, ROR1^P826A^, or ROR1^P841A^ each had high-levels of phosphorylated ERK1/2 that were comparable to that of MEC1-ROR1 cells expressing wild-type ROR1 (WT ROR1) (Fig. 2e). Such levels were higher than that noted for MEC1 cells expressing ROR1^ΔPRD^ or ROR1^P808A^, each of which had low levels of phosphorylated ERK1/2 comparable to that of MEC1 cells lacking expression of ROR1 (Fig. 2e). These results indicate that the PRD, and more specifically the ROR1 proline residue at 808, is critical for the ROR1-associated activation of ERK1/2 in MEC1-ROR1 cells. Moreover, such enhanced activation of ERK1/2 in MEC1-ROR1 cells could be inhibited by anti-Wnt5a antibody or cirmtuzumab (Supplementary Fig. S3A, B).

### Wnt5a-induced activation of ERK1/2 is dependent on DOCK2

**Fig. 3:**
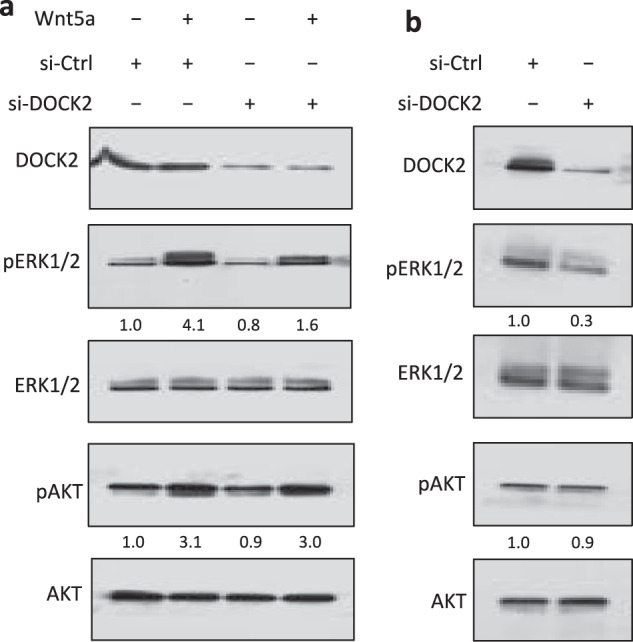
DOCK2 factors in Wnt5a-induced activation of ERK1/2, but not AKT. **a** Immunoblot analysis of lysates prepared from freshly-isolated primary CLL cells (representative of 3 patients) transfected 72 h previously with control siRNA or siRNA targeting DOCK2, then treated without (-) or with (+) Wnt5a; expression of DOCK2, total ERK1/2, AKT, and activated pERK1/2, pAKT was measured, as indicated on the left. The numbers between two lanes are ratios of band IOD of pERK1/2 or pAKT versus total ERK1/2 or AKT respectively, and normalized with respect to that of untreated cells. **b** Immunoblot analysis of lysates prepared from MEC1-ROR1 cells transfected 72 h previously with control siRNA or siRNA targeting DOCK2; expression of DOCK2, total ERK1/2, AKT, and activated pERK1/2, pAKT was measured, as indicated on the left. The numbers between two lanes are ratios of band IOD of pERK1/2 or pAKT versus total ERK1/2 or AKT respectively, and normalized to that of untreated cells.

In prior studies we found that the proline residue at 808 of ROR1 also was required for the Wnt5a-induced docking to ROR1 of the dedicator of cytokinesis protein 2 (DOCK2) [16], a Rac-specific guanine-nucleotide-exchange factor (GEF) [31, 32]. Because of this, we examined whether DOCK2 was necessary for Wnt5a-induced activation of intracellular signaling molecules, such as ERK1/2 or AKT, in primary CLL cells, as well as in MEC1-ROR1. We found that reducing expression of DOCK2 using specific siRNA in primary CLL cells (Fig. 3a, upper panel) impaired the capacity of Wnt5a to induce activation of ERK1/2 (Fig. 3a, middle panels), but not AKT in CLL cells (Fig. 3a, lower panel). Furthermore, we found that reducing expression of DOCK2 with specific siRNA in MEC1-ROR1 (Fig. 3b, upper panel) decreased the levels of phosphorylated ERK1/2 relative to that of MEC1-ROR1 treated with siCtrl (Fig. 3b, middle panels). This again did not repress activation of AKT (Fig. 3b, lower panel), implicating that DOCK2 contributes to ROR1-dependent activation of ERK1/2, but not AKT.

Next we examined whether the capacity of ROR1/DOCK2 to induce activation of ERK1/2 was dependent upon Rac1/2, which are Rho-GTPases that are activated by ROR1/DOCK2 in response to Wnt5a. For this, we treated CLL cells without or with Wnt5a, alone or in combination with EHT1864 (10 μM) [33, 34], a small molecule that can inhibit activated Rac1/2. We noted that the Wnt5a-induced activation of ERK1/2 could be inhibited by EHT1864 (Supplementary Fig. S4A), suggesting that Wnt5a-induced phosphorylation of ERK1/2 required activated Rac1/2. Consistent with this notion we noted that treatment with EHT1864 also could reduce the levels of phosphorylated ERK1/2 in MEC1-ROR1 to levels comparable to those noted in MEC1 cells lacking ROR1 (Supplementary Fig. S4B).

### Wnt5a induces ROR1-dependent tyrosine phosphorylation of DOCK2

**Fig. 4:**
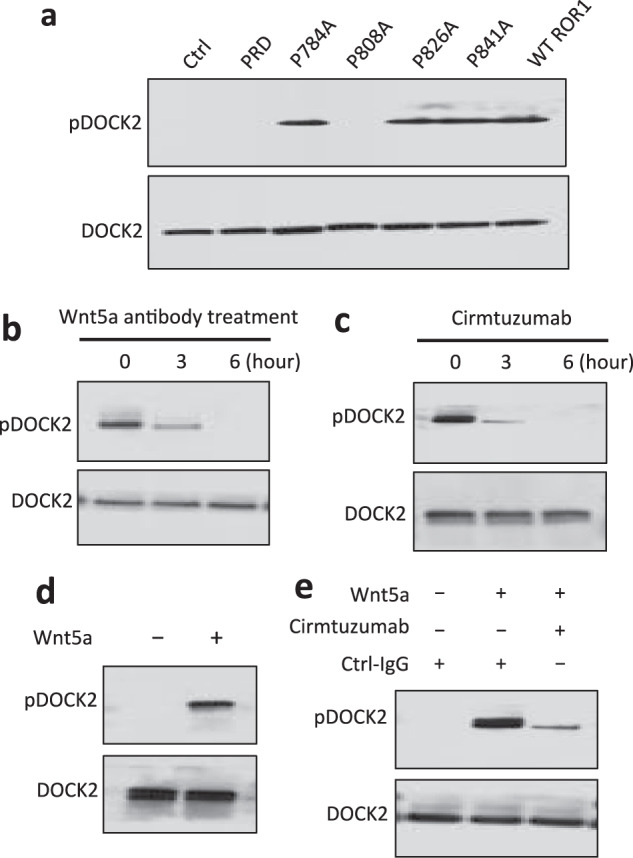
Wnt5a induces ROR1-dependent phosphorylation of DOCK2 in CLL. **a** Tyrosine phosphorylation of DOCK2 was confirmed by immunoblot analysis of anti-DOCK2 immune precipitates (ip), using lysates of MEC1-Ctrl, MEC1-ΔPRD, MEC1-ROR1 (W/T) or MEC1 cells transfected with each of the various mutated forms of ROR1, as indicated on the top; the membranes were probed with anti-DOCK2 or anti-phospho tyrosine antibody (pDOCK2), as indicated on the left. **b** Immunoblot analysis of anti-DOCK2 ip, using lysates prepared from MEC1-ROR1 cells that had been treated with Wnt5a neutralizing antibody (2 µg/ml, R & D) for the times indicated at the top (in hours); membranes were probed with anti-phospho tyrosine (pDOCK2) or anti-DOCK2 antibody, as indicated on the left. **c** Immunoblot analysis of anti-DOCK2 ip, using lysates prepared from MEC1-ROR1 cells that had been treated with cirmtuzumab (20 µg/ml) for the times indicated at the top (in hours); membranes were probed with anti-phospho tyrosine (pDOCK2), anti-DOCK2 antibody, as indicated on the left. **d** Immunoblot analysis of anti-DOCK2 ip, using lysates prepared from overnight, serum-starved primary CLL cells (representative of 3 patients) that subsequently were treated for 5 min without (-) or with (+) Wnt5a (100 ng/ml), as indicated on the top; the membranes were probed with anti-DOCK2 or anti-phospho tyrosine antibody (pDOCK2), as indicated on the left. **e** Immunoblot analysis of anti-DOCK2 ip, using lysates prepared from overnight, serum-starved primary CLL cells (representative of 3 patients) that subsequently were treated with Ctrl-IgG or cirmtuzumab (20 μg/ml), without (-) or with (+) Wnt5a (100 ng/ml), as indicated on the top; the membranes were probed with anti-DOCK2 or anti-phospho tyrosine antibody (pDOCK2), as indicated on the left.

Recent studies suggest that activation of DOCK2 may be associated with tyrosine phosphorylation of this GEF. It recently was shown that CXCL12 stimulation in T lymphocytes, for example, induces phosphorylation of DOCK2, which was associated with chemokine-induced activation of RhoGTPases [35]. To examine whether DOCK2 also was phosphorylated in response to signaling via Wnt5a-ROR1, we examined the anti-DOCK2 i.p. for tyrosine phosphorylated DOCK2 using lysates generated from MEC1 cells, or MEC1 cells made to express wild-type ROR1 or any one of the various ROR1 mutants. We found MEC1-ROR1 cells had phosphorylated DOCK2, as did MEC1 cells expressing ROR1^P784A^, ROR1^P826A^, or ROR1^P841A^ (Fig. 4a). In contrast DOCK2 was not phosphorylated in MEC1 cells or MEC1 expressing ROR1^ΔPRD^ or ROR1^P808A^ (Fig. 4a). These results are consistent with the notion that tyrosine phosphorylation of DOCK2 is dependent upon its capacity to complex with ROR1 in response to autocrine Wnt5a, which induces signaling via ROR1. Consistent with this notion, we found that treatment of MEC1-ROR1 cells with cirmtuzumab or an antibody capable of neutralizing Wnt5a down-modulated the levels of phosphorylated DOCK2 in a time-dependent manner (Fig. 4b, c).

We next examined whether DOCK2 was phosphorylated in primary CLL cells. We performed mass spectrometry based proteomic analysis and found a phospho peptide of DOCK2 at tyrosine (Y) 985 in freshly isolated CLL cells (Supplementary Fig. S5). We also identified that DOCK2 was phosphorylated in freshly isolated CLL cells by immunoblot analysis (Supplementary Fig. S6). However, culture of CLL cells in serum-free media lacking Wnt5a down-modulated the levels of tyrosine phosphorylated DOCK2 in a time-dependent manner (Supplementary Fig. S6). On the other hand, treatment of serum-starved CLL cells with exogenous Wnt5a for 5 min could induce tyrosine phosphorylation of DOCK2 (Fig. 4d). That such phosphorylation was dependent on ROR1-signaling was indicated by our finding that cirmtuzumab, but not a control Ig of irrelevant specificity, could inhibit the capacity of Wnt5a to induce tyrosine-phosphorylation of DOCK2 (Fig. 4e).

### Phosphorylation of ERK1/2 or DOCK2 is higher in CLL cells that express ROR1

**Fig. 5:**
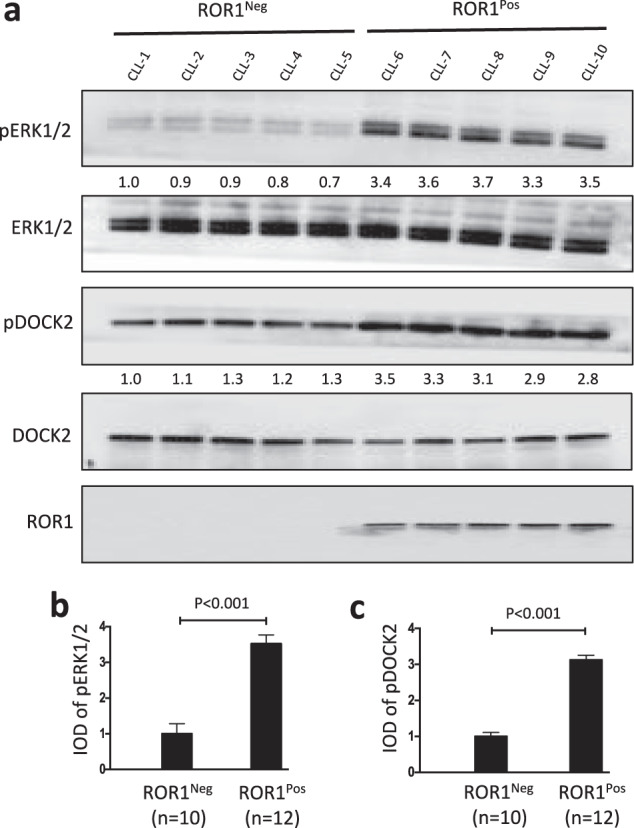
Phosphorylation of ERK1/2 or DOCK2 is high in ROR1^Pos^ CLL. **a** Immunoblot analysis of lysates prepared from primary CLL cells of different ROR1^Pos^ or ROR1^Neg^ patients; membranes were probed with anti-ERK1/2, anti-phospho pERK1/2, as indicated on the left (upper panels). The numbers between two lanes are ratios of band IOD of phosphorylated ERK1/2 relative to total ERK1/2, and normalized with respect to that of CLL-1. Middle panels provide immunoblot analyses of anti-DOCK2 ip, using lysates prepared from ROR1^Pos^ or ROR1^Neg^ CLL cells; membranes were probed with anti-DOCK2, anti-phospho tyrosine (pDOCK2), as indicated on the left (middle panels). The numbers between two lanes are ratios of band IOD of phosphorylated versus total DOCK2, and normalized with respect to that of CLL-1. An immunoblot of the whole-cell lysates probed with anti-ROR1 mAb is provided in the bottom panel. **b** Phosphorylation of ERK1/2 was measured by immunoblot analysis, using lysates prepared from ROR1^Pos^ CLL cells (*N* = 12 patient samples, (8 U-CLL and 4 M-CLL)) or ROR1^Neg^ CLL cells (*n* = 10 patient samples, (3 U-CLL and 7 M-CLL)). The ratios of band IOD of phosphorylated versus total ERK1/2 was determined, normalized to that of CLL-1, and depicted in the graph. Data are shown as mean ± SD. *P* < 0.001, as assessed by 2-tailed Student’s *t* test. **c** Phosphorylation of DOCK2 was measured by immunoblot analysis of anti-DOCK2 ip, using lysates prepared from primary CLL cells of different ROR1^Pos^ (*n* = 12, (8 U-CLL and 4 M-CLL)) or ROR1^Neg^ (*n* = 10, (3 U-CLL and 7 M-CLL)) patients. The ratios of band IOD (integrated optical density) of phosphorylated versus total DOCK2 was determined, normalized to that of CLL-1, and plotted in the graph. Data are shown as mean ± SD. *P* < 0.001, as assessed by 2-tailed Student’s *t* test.

We examined whether the levels of phosphorylated ERK1/2 or DOCK2 in freshly isolated CLL cells was associated with expression of ROR1. Prior studies found heterogeneity in levels at which ROR1 was expressed on CLL cells, and that ~5% of patients had CLL cells with low-to-negligible levels of ROR1 [36]. We examined levels of phosphorylated ERK1/2 or DOCK2 in CLL cells that expressed ROR1 (ROR1^Pos^) versus CLL cells of the small subgroup of patients that did not (termed ROR1^Neg^). We found that ROR1^Neg^ CLL cells that used either U or M IGHV had levels of ERK1/2 or DOCK2 that were comparable to that of ROR1^Pos^ CLL cells (Fig. 5a). However, the mean ratios of phosphorylated to total ERK1/2 or DOCK2 were significantly lower in ROR1^Neg^ CLL cells (*n* = 10) than in ROR1^Pos^ CLL cells (*n* = 12) (*P* < 0.001) (Fig. 5a-c).

### Cirmtuzumab, but not ibrutinib, could inhibit Wnt5a-induced phosphorylation of ERK1/2 or DOCK2

We examined the CLL cells of patients before and after treatment with ibrutinib for activation of ERK1/2. The CLL cells collected from patients treated for 1 month with ibrutinib at 420 mg per day had ratios of phosphorylated ERK1/2 versus total ERK1/2 that were comparable to that detected in the CLL cells of the same patients prior to therapy (Supplementary Fig. S7A, B), suggesting that ibrutinib does not inhibit the constitutive activation of ERK1/2 that apparently is induced by plasma Wnt5a.

**Fig. 6:**
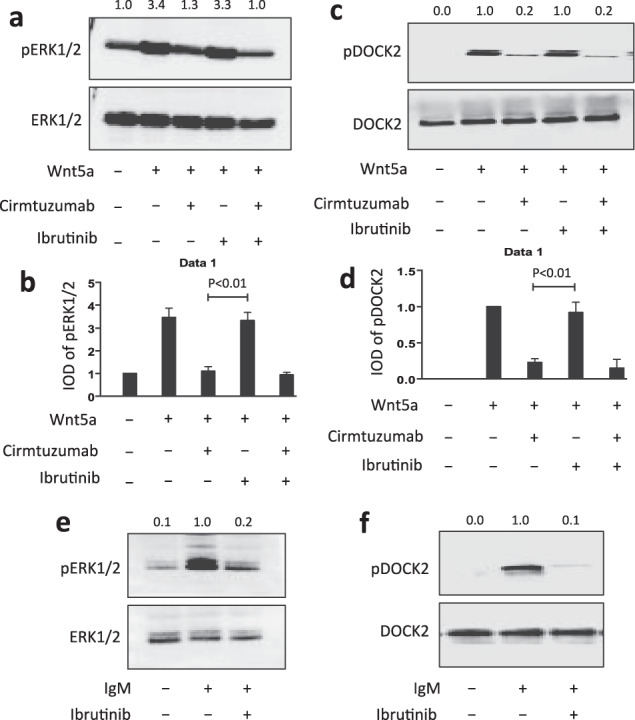
Cirmtuzumab, but not ibrutinib, can inhibit Wnt5a induced activation of ERK1/2 or DOCK2. **a** Immunoblot analysis of lysates prepared from CLL cells cultured overnight in serum-free medium (representative of 3 patients) that subsequently were treated with cirmtuzumab (20 μg/ml) and/or ibrutinib (0.5 μM), without (-) or with (+) Wnt5a (100 ng/ml), as indicated at the bottom; the membranes were probed with anti-ERK1/2 or anti-phospho pERK1/2 antibody, as indicated on the left. The numbers at the top lane are ratios of band IOD of phosphorylated versus total ERK1/2, and normalized with respect to that of untreated samples. **b** Phosphorylation of ERK1/2 was measured in serum-starved CLL cells that subsequently were treated with cirmtuzumab (20 μg/ml) and/or ibrutinib (0.5 μM), without (-) or with (+) Wnt5a (100 ng/ml), as indicated at the bottom. Data are shown as mean ± SD from three independent experiments, (*n* = 3). *P* < 0.01, as determined by 2-tailed Student’s *t* test. **c** Immunoblot analysis of anti-DOCK2 ip, using lysates prepared from CLL cells cultured overnight in serum-free medium (representative of 3 patients) that subsequently were treated with cirmtuzumab (20 μg/ml) and/or ibrutinib (0.5 μM), without (-) or with (+) Wnt5a (100 ng/ml), as indicated at the bottom; the membranes were probed with anti-DOCK2 or anti-phospho tyrosine antibody (pDOCK2), as indicated on the left. The numbers at the top lane are ratios of band IOD of phosphorylated versus total DOCK2, and normalized with respect to that of untreated samples. **d** Phosphorylation of DOCK2 was measured in CLL cells cultured overnight in serum-free medium and subsequently treated with cirmtuzumab (20 μg/ml) and/or ibrutinib (0.5 μM), without (-) or with (+) Wnt5a (100 ng/ml), as indicated at the bottom. Data are shown as mean ± SD from three independent experiments, (*n* = 3). *P* < 0.01, as determined by 2-tailed Student’s *t* test. **e** Immunoblot analysis of lysates prepared from CLL cells cultured overnight in serum-free medium (representative of 3 patients) that subsequently were treated with ibrutinib (0.5 μM), without (-) or with (+) anti-human IgM F(ab)_2_ (10 μg/ml), as indicated at the bottom; the membranes were probed with anti-ERK1/2 or anti-phospho pERK1/2 antibody, as indicated on the left. The numbers at the top lane are ratios of band IOD of phosphorylated versus total ERK1/2, and normalized to that of untreated samples. **f** Immunoblot analysis of anti-DOCK2 ip, using lysates prepared from CLL cells cultured overnight in serum-free medium (representative of 3 patients) that subsequently were treated with ibrutinib (0.5 μM), without (-) or with (+) anti-human IgM F(ab)_2_ (10 μg/ml), as indicated at the bottom; the membranes were probed with anti-DOCK2 or anti-phospho tyrosine antibody (pDOCK2), as indicated on the left. The numbers at the top lane are ratios of band IOD of phosphorylated versus total DOCK2, and normalized to that of untreated samples.

To test this directly, we examined whether ibrutinib could inhibit the phosphorylation of ERK1/2 or DOCK2 induced by Wnt5a in serum-starved CLL cells. In contrast to treatment with cirmtuzumab, we found that ibrutinib could not inhibit Wnt5a-induced phosphorylation of ERK1/2 or DOCK2 (Fig. 6a-d). Nonetheless, the concentration of ibrutinib used in these studies was sufficient to block BCR-signaling, as this same concentration (0.5 µM) was sufficient to inhibit the capacity of anti-µ to induce phosphorylation of ERK1/2 or DOCK2 in CLL cells of the same patient sample (Fig. 6e, f).

## Discussion

In the present study we demonstrated that Wnt5a can induce phosphorylation of ERK1/2 in CLL cells via a ROR1/DOCK2-dependent pathway. Inhibition of ERK1/2 using a small molecule inhibitor or ERK1/2-specific siRNA inhibited the capacity of Wnt5a to enhance of CLL-cell proliferation, which also could be inhibited by cirmtuzumab, demonstrating that the capacity of Wnt5a to enhance CLL-cell proliferation is dependent on ROR1 and ERK1/2.

ERK1/2 is an integral member of the mitogen-activated protein kinase (MAPK) signaling pathway. Phosphorylation of ERK1/2 allows for its translocation from the cytosol to the nucleus to activate transcription factors, such as Jun, Fos, Ets-1, Elk, HIF-1, and cyclic AMP receptor binding protein (CREB), which collectively enhance CLL-cell proliferation and survival [3, 37, 38]. We found that the genes induced by such factors are significantly reduced in CLL cells of patients treated with cirmtuzumab from levels noted prior to therapy, indicating that cirmtuzumab can repress expression of genes induced by activation of ERK1/2 in vivo.

Prior studies using the leukemia cell-line, MEC1, found that these cells expressed Wnt5a [10], but not ROR1 [29]. We found that MEC1 cells transfected to express ROR1 (MEC1-ROR1) had higher levels of phosphorylated ERK1/2 than parental MEC1 cells and that the levels of ERK1/2 activation in MEC1-ROR1 could be repressed by treatment of these cells with neutralizing antibodies to Wnt5a or cirmtuzumab, indicating that constitutive high-level activation of ERK1/2 in MEC1-ROR1 was due to the capacity of endogenous Wnt5a to act as an autocrine to induce ROR1-signaling. This allowed us to perform structure-function relationship studies on ERK1/2 activation using MEC1 cells made to express ROR1 or various mutant forms of ROR1. We discovered that MEC1 cells expressing ROR1^ΔPRD^, lacking the PRD, and more specifically MEC1-ROR^P808A^, lacking the proline residue at position 808 within the PRD, had levels of phospho-ERK1/2 that were comparable to that of MEC1 cells, despite expressing comparable amounts of the ROR1. Because our prior studies had determined that proline at position 808 to be necessary for ROR1 to interact with DOCK2 in response to Wnt5a, we deduced that DOCK2 was required for activation of ERK1/2 in response to Wnt5a. Consistent with this notion, we found that repressing expression of DOCK2 using specific siRNA in MEC1-ROR1 cells, or in CLL cells, repressed the capacity of Wnt5a to induce ERK1/2 phosphorylation.

DOCK2 is a guanine exchange factor (GEF) that is expressed predominately by hematopoietic cells, in which it governs activation, migration, and proliferation through activation of Rac1/2 [39]. The SH3 domain of DOCK2 is thought to allow it to interact with the PRD of ROR1 and/or the carboxyl terminal of engulfment and cell motility factor 1 (Elmo1), which mitigates ubiquitination of DOCK2 [16, 40]. The carboxyl-terminal polyamino-acid (PAA) region of DOCK2 also enhances its accumulation upon binding phosphatidic acid, which can be blocked by a small molecule inhibitor [41]. DOCK2 also has an evolutionarily conserved Dock homology region (DHR) domain, DHR-1, which can bind phosphatidylinositol 3,4,5-triphosphate (PIP3), promoting its translocation to the leading edge of the cell upon activation. Upon activation, the DHR-2 domain of DOCK2 activates Rac1 and Rac2 [42]. Here we report that DOCK2 activation in CLL in response to anti–µ or Wnt5a also is associated with its tyrosine phosphorylation, as noted in recent studies on T lymphocytes stimulated with chemokine [35]. Currently, it was unclear whether phosphorylation of DOCK2 is required for its capacity to activate Rac1/2 with subsequent activation of ERK1/2. However, the results of this study shows that DOCK2 phosphorylation in response to Wnt5a associates with its activation, which requires its complexing with ROR1. Such Wnt5a-induced activation could be inhibited by cirmtuzumab, but not by ibrutinib, which instead could inhibit the phosphorylation/activation of DOCK2 in response to anti-µ. Because the lymphocytes of healthy adults do not express ROR1 [13], this pathway of Wnt5a-induced DOCK2 phosphorylation may be restricted to leukemia B cells.

Similarly, activation of signaling molecules other than ROR1 can induce phosphorylation of ERK1/2 in lymphocytes or CLL cells. Notably, stimulation afforded by activation of the BCR or chemokine-receptors can induce phosphorylation of ERK1/2 and thereby potentially enhance CLL-cell proliferation [3, 4, 43]. The capacity of such signaling molecules to affect phosphorylation ERK1/2 and leukemia-cell proliferation, however, is dependent on kinases such as BTK, which can be inhibited by ibrutinib [44, 45], potentially accounting for the noted clinical activity of this drug in the treatment of patients with CLL. However, the noted inability of ibrutinib to block ERK1/2 activation or effect clearance of CLL cells in almost all patients treated with this drug [46], suggests that other signaling pathways sustain the survival/growth of CLL cells of patients undergoing therapy with inhibitors of BTK. The ROR1/DOCK2-signaling pathway may be important in this regard.

Previously we described that ROR1 also could associate with other cytoskeleton proteins such as HS1 or cortactin [19, 47]. In such cases, HS1/cortactin binds to the SH3-binding site, which is dependent upon the proline at position 841 of ROR1, and activates RhoA to enhance the migration-capacity of CLL cells. In contrast, DOCK2 associates with the SH3-binding site that is dependent upon the proline at position 808 of ROR1 and appears unaffected by the 841-position proline-to-alanine substitution, which can impair the capacity of ROR1 to activate RhoA or enhance the migration capacity of CLL cells [19, 47], indicating that DOCK2 is not required for the capacity of Wnt5a to enhance chemokine-directed migration via activation of ROR1.

In summary, we found that Wnt5a induces ROR1 to recruit and activate DOCK2, leading to activation of ERK1/2, which appears responsible for the capacity of Wnt5a to enhance leukemia-cell proliferation. This pathway may be blocked by cirmtuzumab, a first-in-class humanized anti-ROR1 mAb, which is being evaluated in patients with CLL or MCL (https://clinicaltrials.gov/ct2/show/NCT02222688) [11, 48]. The capacity of cirmtuzumab to inhibit Wnt5a-induced ROR1-signaling, which appears active in CLL cells of patients treated with ibrutinib [46], provides rationale for the clinical evaluation of this antibody, alone or in combination with ibrutinib or other targeted therapies, for patients with CLL or other ROR1-expressing malignancies, such as MCL [49].

## Supplementary information

Supplemental Information

Supplemental Figures

## References

[1] Zhang S, Kipps TJ (2014). The pathogenesis of chronic lymphocytic leukemia. Annu Rev Pathol.

[2] Kipps TJ, Stevenson FK, Wu CJ, Croce CM, Packham G, Wierda WG (2017). Chronic lymphocytic leukaemia. Nat Rev Dis Prim.

[3] Yaktapour N, Meiss F, Mastroianni J, Zenz T, Andrlova H, Mathew NR (2014). BRAF inhibitor-associated ERK activation drives development of chronic lymphocytic leukemia. J Clin Investig.

[4] Shukla A, Rai K, Shukla V, Chaturvedi NK, Bociek RG, Pirruccello SJ (2016). Sprouty 2: a novel attenuator of B-cell receptor and MAPK-Erk signaling in CLL. Blood.

[5] Apollonio B, Scielzo C, Bertilaccio MT, Ten Hacken E, Scarfo L, Ranghetti P (2013). Targeting B-cell anergy in chronic lymphocytic leukemia. Blood.

[6] O’Hayre M, Salanga CL, Kipps TJ, Messmer D, Dorrestein PC, Handel TM (2010). Elucidating the CXCL12/CXCR4 signaling network in chronic lymphocytic leukemia through phosphoproteomics analysis. PLoS ONE.

[7] Chen SS, Chang BY, Chang S, Tong T, Ham S, Sherry B (2016). BTK inhibition results in impaired CXCR4 chemokine receptor surface expression, signaling and function in chronic lymphocytic leukemia. Leukemia.

[8] Crassini K, Stevenson WS, Mulligan SP, Best OG (2015). The MEK1/2 inhibitor, MEKi-1, induces cell death in chronic lymphocytic leukemia cells under conditions that mimic the tumor microenvironment and is synergistic with fludarabine. Leuk Lymphoma.

[9] Garaud S, Taher TE, Debant M, Burgos M, Melayah S, Berthou C (2018). CD5 expression promotes IL-10 production through activation of the MAPK/Erk pathway and upregulation of TRPC1 channels in B lymphocytes. Cell Mol Immunol.

[10] Yu J, Chen L, Cui B, Widhopf GF, Shen Z, Wu R (2016). Wnt5a induces ROR1/ROR2 heterooligomerization to enhance leukemia chemotaxis and proliferation. J Clin Investig.

[11] Choi MY, Widhopf GF, Ghia EM, Kidwell RL, Hasan MK, Yu J (2018). Phase I Trial: cirmtuzumab Inhibits ROR1 Signaling and Stemness Signatures in Patients with Chronic Lymphocytic Leukemia. Cell Stem Cell.

[12] Wang X, Zhao X, Yi Z, Ma B, Wang H, Pu Y (2018). WNT5A promotes migration and invasion of human osteosarcoma cells via SRC/ERK/MMP-14 pathway. Cell Biol Int.

[13] Fukuda T, Chen L, Endo T, Tang L, Lu D, Castro JE (2008). Antisera induced by infusions of autologous Ad-CD154-leukemia B cells identify ROR1 as an oncofetal antigen and receptor for Wnt5a. Proc Natl Acad Sci USA.

[14] Baskar S, Kwong KY, Hofer T, Levy JM, Kennedy MG, Lee E (2008). Unique cell surface expression of receptor tyrosine kinase ROR1 in human B-cell chronic lymphocytic leukemia. Clin Cancer Res.

[15] Daneshmanesh AH, Mikaelsson E, Jeddi-Tehrani M, Bayat AA, Ghods R, Ostadkarampour M (2008). Ror1, a cell surface receptor tyrosine kinase is expressed in chronic lymphocytic leukemia and may serve as a putative target for therapy. Int J Cancer.

[16] Hasan MK, Yu J, Widhopf GF, Rassenti LZ, Chen L, Shen Z (2018). Wnt5a induces ROR1 to recruit DOCK2 to activate Rac1/2 in chronic lymphocytic leukemia. Blood.

[17] Zhang Q, Wang HY, Liu X, Nunez-Cruz S, Jillab M, Melnikov O (2019). Cutting Edge: ROR1/CD19 Receptor Complex Promotes Growth of Mantle Cell Lymphoma Cells Independently of the B Cell Receptor-BTK Signaling Pathway. J Immunol.

[18] Widhopf GF, Cui B, Ghia EM, Chen L, Messer K, Shen Z (2014). ROR1 can interact with TCL1 and enhance leukemogenesis in Emu-TCL1 transgenic mice. Proc Natl Acad Sci USA.

[19] Hasan MK, Yu J, Chen L, Cui B, Widhopf Ii GF, Rassenti L (2017). Wnt5a induces ROR1 to complex with HS1 to enhance migration of chronic lymphocytic leukemia cells. Leukemia.

[20] Subramanian A, Tamayo P, Mootha VK, Mukherjee S, Ebert BL, Gillette MA (2005). Gene set enrichment analysis: a knowledge-based approach for interpreting genome-wide expression profiles. Proc Natl Acad Sci USA.

[21] Roskoski R (2012). ERK1/2 MAP kinases: structure, function, and regulation. Pharm Res.

[22] Dittmer J (2003). The biology of the Ets1 proto-oncogene. Mol Cancer.

[23] Dimova EY, Moller U, Herzig S, Fink T, Zachar V, Ebbesen P (2005). Transcriptional regulation of plasminogen activator inhibitor-1 expression by insulin-like growth factor-1 via MAP kinases and hypoxia-inducible factor-1 in HepG2 cells. Thromb Haemost.

[24] Adiseshaiah P, Li J, Vaz M, Kalvakolanu DV, Reddy SP (2008). ERK signaling regulates tumor promoter induced c-Jun recruitment at the Fra-1 promoter. Biochem Biophys Res Commun.

[25] Ashok C, Owais S, Srijyothi L, Selvam M, Ponne S, Baluchamy S (2019). A feedback regulation of CREB activation through the CUL4A and ERK signaling. Med Oncol.

[26] Chaikuad A, Tacconi EM, Zimmer J, Liang Y, Gray NS, Tarsounas M (2014). A unique inhibitor binding site in ERK1/2 is associated with slow binding kinetics. Nat Chem Biol.

[27] Morris EJ, Jha S, Restaino CR, Dayananth P, Zhu H, Cooper A (2013). Discovery of a novel ERK inhibitor with activity in models of acquired resistance to BRAF and MEK inhibitors. Cancer Disco.

[28] Stacchini A, Aragno M, Vallario A, Alfarano A, Circosta P, Gottardi D (1999). MEC1 and MEC2: two new cell lines derived from B-chronic lymphocytic leukaemia in prolymphocytoid transformation. Leuk Res.

[29] Zhang S, Chen L, Wang-Rodriguez J, Zhang L, Cui B, Frankel W (2012). The onco-embryonic antigen ROR1 is expressed by a variety of human cancers. Am J Pathol.

[30] Yu J, Chen L, Cui B, Widhopf GF, Shen Z, Wu R (2015). Wnt5a induces ROR1/ROR2 heterooligomerization to enhance leukemia chemotaxis and proliferation. J Clin Investig.

[31] Nishihara H, Kobayashi S, Hashimoto Y, Ohba F, Mochizuki N, Kurata T (1999). Non-adherent cell-specific expression of DOCK2, a member of the human CDM-family proteins. Biochim Biophys Acta.

[32] Cote JF, Vuori K (2002). Identification of an evolutionarily conserved superfamily of DOCK180-related proteins with guanine nucleotide exchange activity. J Cell Sci.

[33] Shutes A, Onesto C, Picard V, Leblond B, Schweighoffer F, Der CJ (2007). Specificity and mechanism of action of EHT 1864, a novel small molecule inhibitor of Rac family small GTPases. J Biol Chem.

[34] Li C, MacDonald JI, Talebian A, Leuenberger J, Seah C, Pasternak SH (2016). Unravelling the Mechanism of TrkA-Induced Cell Death by Macropinocytosis in Medulloblastoma Daoy Cells. Mol Cell Biol.

[35] Toffali L, Montresor A, Mirenda M, Scita G, Laudanna C (2017). SOS1, ARHGEF1, and DOCK2 rho-GEFs Mediate JAK-Dependent LFA-1 Activation by Chemokines. J Immunol.

[36] Cui B, Ghia EM, Chen L, Rassenti LZ, DeBoever C, Widhopf GF (2016). High-level ROR1 associates with accelerated disease progression in chronic lymphocytic leukemia. Blood.

[37] Chang F, Steelman LS, Lee JT, Shelton JG, Navolanic PM, Blalock WL (2003). Signal transduction mediated by the Ras/Raf/MEK/ERK pathway from cytokine receptors to transcription factors: potential targeting for therapeutic intervention. Leukemia.

[38] Zhang W, Liu HT (2002). MAPK signal pathways in the regulation of cell proliferation in mammalian cells. Cell Res.

[39] Chen Y, Meng F, Wang B, He L, Liu Y, Liu Z (2018). Dock2 in the development of inflammation and cancer. Eur J Immunol.

[40] Sanui T, Inayoshi A, Noda M, Iwata E, Stein JV, Sasazuki T (2003). DOCK2 regulates Rac activation and cytoskeletal reorganization through interaction with ELMO1. Blood.

[41] Nishikimi A, Fukuhara H, Su W, Hongu T, Takasuga S, Mihara H (2009). Sequential regulation of DOCK2 dynamics by two phospholipids during neutrophil chemotaxis. Science.

[42] Nishikimi A, Uruno T, Duan X, Cao Q, Okamura Y, Saitoh T (2012). Blockade of inflammatory responses by a small-molecule inhibitor of the Rac activator DOCK2. Chem Biol.

[43] Burger JA, Tsukada N, Burger M, Zvaifler NJ, Dell’Aquila M, Kipps TJ (2000). Blood-derived nurse-like cells protect chronic lymphocytic leukemia B cells from spontaneous apoptosis through stromal cell-derived factor-1. Blood.

[44] Cheng S, Ma J, Guo A, Lu P, Leonard JP, Coleman M (2014). BTK inhibition targets in vivo CLL proliferation through its effects on B-cell receptor signaling activity. Leukemia.

[45] Ten Hacken E, Sivina M, Kim E, O’Brien S, Wierda WG, Ferrajoli A (2016). Functional Differences between IgM and IgD Signaling in Chronic Lymphocytic Leukemia. J Immunol.

[46] Yu J, Chen L, Cui B, Wu C, Choi MY, Chen Y (2017). Cirmtuzumab inhibits Wnt5a-induced Rac1 activation in chronic lymphocytic leukemia treated with ibrutinib. Leukemia.

[47] Hasan MK, Rassenti L, Widhopf GF, Yu J, Kipps TJ (2019). Wnt5a causes ROR1 to complex and activate cortactin to enhance migration of chronic lymphocytic leukemia cells. Leukemia.

[48] Choi MY, Widhopf GF, Wu CC, Cui B, Lao F, Sadarangani A (2015). Pre-clinical Specificity and Safety of UC-961, a First-In-Class Monoclonal Antibody Targeting ROR1. Clin Lymphoma Myeloma Leuk.

[49] Yu J, Chen Y, Chen L, Zhang L, Rassenti LZ, Widhopf GF (2018). Cirmtuzumab inhibits ibrutinib-resistant, Wnt5a-induced Rac1 activation and proliferation in mantle cell lymphoma. Oncotarget.

