# Supplementary material for: Wnt5a enhances proliferation of chronic lymphocytic leukemia and ERK1/2 phosphorylation via a ROR1/DOCK2-dependent mechanism
Source: Leukemia. 2020 Oct 23;35(6):1621–30. doi: 10.1038/s41375-020-01055-7 (PMC8062590; doi:10.1038/s41375-020-01055-7)
Supplement: Supplementary file 1 — Supplemental Information [file 41375_2020_1055_MOESM1_ESM.docx]

**supplementary information**

Wnt5a enhances proliferation of chronic lymphocytic leukemia and ERK1/2 phosphorylation via a ROR1/DOCK2-dependent mechanism

**Supplemental Materials and Methods**

**CLL Specimens and Cell Culture**

Blood samples were collected from CLL patients at the Moores Cancer Center. Peripheral blood mononuclear cells (PBMC) were isolated by density centrifugation using Ficoll-Paque PLUS (GE Healthcare Life Sciences), suspended in 10% DMSO (Sigma-Aldrich) and 90% FBS (Omega Scientific) and stored in liquid nitrogen. MEC1 cells were cultured in 1% Penicillin/Streptomycin, 10% FBS containing RPMI-1640 medium, and maintained at 37°C in a humidified atmosphere of 5% CO_2_ at 37 ̊C, and tested negative for mycoplasma contamination. Supplements and media were purchased from Life Technologies (Carlsbad, CA, USA).

**Immunoprecipitation analysis**

Immunoprecipitation analysis was performed as described.^1, 2^ Cells were lysed in a buffer containing 1% Nonidet P-40, 10 mM Tris-HCl (pH 7.5), 50 mM NaCl, and 1 mM EDTA with protease inhibitors (Roche). The lysates were cleared by centrifugation at 16,000 x *g* for 15 minutes. Immune precipitates were isolated using protein A agarose beads, followed by immunoblot analysis. The anti-DOCK2 antibody for immune precipitation was obtained from Santa Cruz Biotechnology, Dallas, TX, USA.

**Cell Proliferation Assay**

The CLL-cell proliferation assay was performed as described.^3, 4^ CLL cells were labeled by carboxyfluorescein succinimidyl ester (CFSE, Life Technologies) and plated at 1.5x10^6^/well/ml in a 24-well tray on a layer of irradiated CD154-expressing HeLa cells (HeLa_CD154_ cells) (80 Gy) at a CLL/HeLa_CD154_ cell ratio of 15:1 in complete RPMI-1640 medium supplemented with 5 ng/ml of recombinant human IL-4 (R&D Systems) and 15 ng/ml of recombinant human IL-10 (R&D Systems). Wnt5a (200 ng/ml, R&D Systems) was added, as indicated in the text. CFSE-labeled CLL cells were analyzed by flow cytometry; Modfit LT software (version 3.0, Verity Software House) was used for analysis of cell proliferation.

**Nucleofection of siRNA and plasmids**

Human B Cell Nucleofector Kit for siRNA or plasmid transfection was from Lonza. CLL cells or MEC1 cells (5 × 10^6^) were suspended in 100 μl Nucleofector Solution with siRNA (Life Technologies) or plasmids (pcDNA3.1 vector expressing human ROR1) and transfected with the Nucleofector II device (program U-015). The transfected cells were cultured in 12-well plates in complete medium for 72 hours and then subjected to immunoblot analysis. Endofree Plasmid Maxi Kits (QIAGEN) were used to purify plasmids for transfection. G418 (1.5 mg/ml) was used for selection of stable MEC1 transfectants, which then were examined via flow cytometry or western blot.

**Site-specific mutation**

We performed site-specific mutations as described previously.^5^ In brief, mutation constructs were generated on the basis of the parental construct (wild-type ROR1) with QuikChange Site-Directed Mutagenesis System (Invitrogen), according to the manufacturer's instructions. The mutations for each construct were verified by DNA sequencing.

The following primer sets were used:

P(784)A, 5'-CAGTGAGTAATCTCAGTAACGCCAGATATC-3' (sense) and 5'-CATGTAATTAGGATATCTGGCGTTACTGAG-3' (antisense);

P(808)A, 5'-GATTGCTGGTTTCATTGGCGCGCCAATACC-3' (sense) and 5'-GGTTCTGAGGTATTGGCGCGCCAATGAAACC-3' (antisense);

P(826)A 5'-CAATGGATACCCAATACCTGCTGGATATGCAGC-3' (sense) and 5'-GGAAACGCTGCATATCCAGCAGGTATTGG-3' (antisense);

P(841)A, 5-CCAGCCAACAGGTGCTCCCAGAGTGATTC-3 (sense) and 5-GCTGAATCACTCTGGGAGCACCTGTTGG-3 (antisense).

**Mass Spectrometry**

MS analysis was performed as described previously.^6, 7^ Briefly, freshly isolated CLL cells were lysed and performed ip using anti-DOCK2 antibody, and subjected to run SDS-PAGE gel, then stained the gel with coomassie solution, and cut the gel at the appropriate DOCK2 band size for phospho proteomics analysis.

**Sample preparation:** Protein samples were diluted in TNE (50 mM Tris pH 8.0, 100 mM NaCl, 1 mM EDTA) buffer. RapiGest SF reagent (Waters Corp.) was added to the mix to a final concentration of 0.1% and samples were boiled for 5 min. TCEP (Tris (2-carboxyethyl) phosphine) was added to 1 mM (final concentration) and the samples were incubated at 37°C for 30 min. Subsequently, the samples were carboxymethylated with 0.5 mg/ml of iodoacetamide for 30 min at 37°C followed by neutralization with 2 mM TCEP (final concentration). Proteins samples prepared as above were digested with trypsin (trypsin:protein ratio - 1:50) overnight at 37°C. RapiGest was degraded and removed by treating the samples with 250 mM HCl at 37°C for 1 h followed by centrifugation at 14000 rpm for 30 min at 4°C. The soluble fraction was then added to a new tube and the peptides were extracted and desalted using C18 desalting columns (Thermo Scientific, PI-87782). Peptides were quantified using BCA assay and a total of 1 ug of peptides were injected for LC-MS analysis.

**LC-MS-MS**: Trypsin-digested peptides were analyzed by ultra high-pressure liquid chromatography (UPLC) coupled with tandem mass spectroscopy (LC-MS/MS) using nano-spray ionization. The nanospray ionization experiments were performed using a Orbitrap fusion Lumos hybrid mass spectrometer (Thermo) interfaced with nano-scale reversed-phase UPLC (Thermo Dionex UltiMate™ 3000 RSLC nano System) using a 25 cm, 75-micron ID glass capillary packed with 1.7-µm C18 (130) BEH^TM^ beads (Waters corporation).  Peptides were eluted from the C18 column into the mass spectrometer using a linear gradient (5–80%) of ACN (Acetonitrile) at a flow rate of 375 μl/min for 1h. The buffers used to create the ACN gradient were: Buffer A (98% H_2_O, 2% ACN, 0.1% formic acid) and Buffer B (100% ACN, 0.1% formic acid). Mass spectrometer parameters are as follows; an MS1 survey scan using the orbitrap detector (mass range (m/z): 400-1500 (using quadrupole isolation), 120000 resolution setting, spray voltage of 2200 V, Ion transfer tube temperature of 275 C, AGC target of 400000, and maximum injection time of 50 ms) was followed by data dependent scans (top speed for most intense ions, with charge state set to only include +2-5 ions, and 5 second exclusion time, while selecting ions with minimal intensities of 50000 at in which the collision event was carried out in the high energy collision cell (HCD Collision Energy of 30%), and the fragment masses where analyzed in the ion trap mass analyzer (With ion trap scan rate of turbo, first mass m/z was 100, AGC Target 5000 and maximum injection time of 35ms). Protein identification and label free quantification was carried out using Peaks Studio 8.5 (Bioinformatics solutions Inc.)

**Statistical analysis**

Data are presented as mean ± SD. Differences between 2 groups were determined by unpaired 2-tailed Student’s *t* test. *P* values of less than 0.05 were considered significant. Analysis for significance was performed with GraphPad Prism 6.0 (GraphPad Software Inc.).

**Supplemental Figure Legends**

**Supplemental Figure S1.** Enrichment plots of gene sets targeted by transcription factors namely genes targeted by E-twenty-six (Ets-1), E-26-oncogene (Elk), Hypoxia-inducible factor-1 (HIF-1), Jun, CREB, or Fos on pre-treatment (Pre-Rx) versus paired post-cirmtuzumab CLL cells at D28 (D28) of patients in phase I clinical trial (N=3). For each gene set, gene-set size (SIZE), normalized enrichment score (NES), and FDR q value (FDR q) are indicated.

**Supplementary Figure S2.** Fluorescence of MEC1 cells, or MEC1 cells transfected with vectors encoding W/T ROR1, truncated ROR1 (ΔPRD), or point-mutated ROR1 after staining with a fluorochrome-labeled isotype control mAb (open histograms) or anti-ROR1 4A5- Alexa-647 mAb (shaded histograms).

**Supplementary Figure S3.** (**A**) Immunoblot analysis of lysates prepared from MEC1-ROR1 cells treated with Ctrl-IgG or anti-Wnt5a antibody (2 µg/ml, R & D) for 6 hours (representative of 2 independent experiments); expression of total ERK1/2, and activated pERK1/2 was measured, as indicated on the left. The numbers at the top lane are ratios of band IOD of pERK1/2 versus total ERK1/2. (**B**) Immunoblot analysis of lysates prepared from MEC1-ROR1 cells treated with Ctrl-IgG or cirmtuzumab (20 μg/ml) for 6 hours (representative of 2 independent experiments); expression of total ERK1/2, and activated pERK1/2 was measured, as indicated on the left. The numbers at the top lane are ratios of band IOD of pERK1/2 versus total ERK1/2.

**Supplemental Figure S4.** *Wnt5a induced activated Rac contributes enhanced activation of ERK1/2*

**(A)** Immunoblot analysis of lysates prepared from primary CLL cells treated without (-) or with (+) Rac inhibitor (100 nM, R&D) and Wnt5a; expression of total ERK1/2, and activated pERK1/2 was measured, as indicated on the left. (**B**) Immunoblot analysis of lysates prepared from MEC1-ROR1 cells treated without (-) or with (+) Rac inhibitor; expression of total ERK1/2, and activated pERK1/2 was measured, as indicated on the left.

**Supplemental Figure S5.** DOCK2 phospho peptide at tyrosine (Y) 985 identified by 2D-nanoLC–MS/MS in anti-DOCK2 immune precipitates (ip) of lysates of freshly isolated CLL cells.

**Supplemental Figure S6.** Immunoblot analysis of anti-DOCK2 ip, using lysates prepared from freshly-isolated primary CLL cells that were serum-starved for the times indicated on the top (in hours); the membranes were probed with anti-DOCK2, or anti-phospho tyrosine antibody (pDOCK2), as indicated on the left.

**Supplemental Figure S7.** (**A**) Immunoblot analysis of lysates prepared from different primary CLL cells derived from CLL patients including pre-treatment (Pre-Rx) or post-treatment of ibrutinib at one month, and developed resistance to ibrutinib therapy; membranes were probed with anti-ERK1/2, anti-phospho pERK1/2, as indicated on the left (upper panels). The numbers between two lanes are ratios of band IOD (integrated optical density) of phosphorylated versus total ERK1/2, normalized with respect to that of pre-treatment. An immunoblot of the whole-cell lysates probed with anti-ROR1 mAb is provided in the bottom panel. (**B**) Phosphorylation of ERK1/2 was measured by immunoblot analysis, using lysates prepared from primary CLL cells derived from CLL patients (n=7) including pre-treatment (Pre-Rx) and post-treatment of ibrutinib at one month. The ratios of band IOD (integrated optical density) of phosphorylated versus total ERK1/2 was determined, normalized to that of pre-treatment, and plotted in the graph. Data are shown as mean ± SD. N.S. indicates not significant, as assessed by 2-tailed Student’s *t* test.

**Supplementary References**

1. Widhopf GF, 2nd, Cui B, Ghia EM, Chen L, Messer K, Shen Z*, et al.* ROR1 can interact with TCL1 and enhance leukemogenesis in Emu-TCL1 transgenic mice. *Proc Natl Acad Sci U S A* 2014 Jan 14; **111**(2)**:** 793-798.

2. Hasan MK, Yu J, Widhopf GF, 2nd, Rassenti LZ, Chen L, Shen Z*, et al.* Wnt5a induces ROR1 to recruit DOCK2 to activate Rac1/2 in chronic lymphocytic leukemia. *Blood* 2018 Apr 20.

3. Yu J, Chen L, Cui B, Widhopf GF, 2nd, Shen Z, Wu R*, et al.* Wnt5a induces ROR1/ROR2 heterooligomerization to enhance leukemia chemotaxis and proliferation. *J Clin Invest* 2016 Feb; **126**(2)**:** 585-598.

4. Fecteau JF, Corral LG, Ghia EM, Gaidarova S, Futalan D, Bharati IS*, et al.* Lenalidomide inhibits the proliferation of CLL cells via a cereblon/p21(WAF1/Cip1)-dependent mechanism independent of functional p53. *Blood* 2014 Sep 04; **124**(10)**:** 1637-1644.

5. Hasan MK, Nafady A, Takatori A, Kishida S, Ohira M, Suenaga Y*, et al.* ALK is a MYCN target gene and regulates cell migration and invasion in neuroblastoma. *Sci Rep* 2013 Dec 20; **3:** 3450.

6. Guttman M, Betts GN, Barnes H, Ghassemian M, van der Geer P, Komives EA. Interactions of the NPXY microdomains of the low density lipoprotein receptor-related protein 1. *Proteomics* 2009 Nov; **9**(22)**:** 5016-5028.

7. McCormack AL, Schieltz DM, Goode B, Yang S, Barnes G, Drubin D*, et al.* Direct analysis and identification of proteins in mixtures by LC/MS/MS and database searching at the low-femtomole level. *Anal Chem* 1997 Feb 15; **69**(4)**:** 767-776.
