# Supplementary material for: Wnt5a enhances proliferation of chronic lymphocytic leukemia and ERK1/2 phosphorylation via a ROR1/DOCK2-dependent mechanism
Source: Leukemia. 2020 Oct 23;35(6):1621–30. doi: 10.1038/s41375-020-01055-7 (PMC8062590; doi:10.1038/s41375-020-01055-7)

Supplementary Figure S1

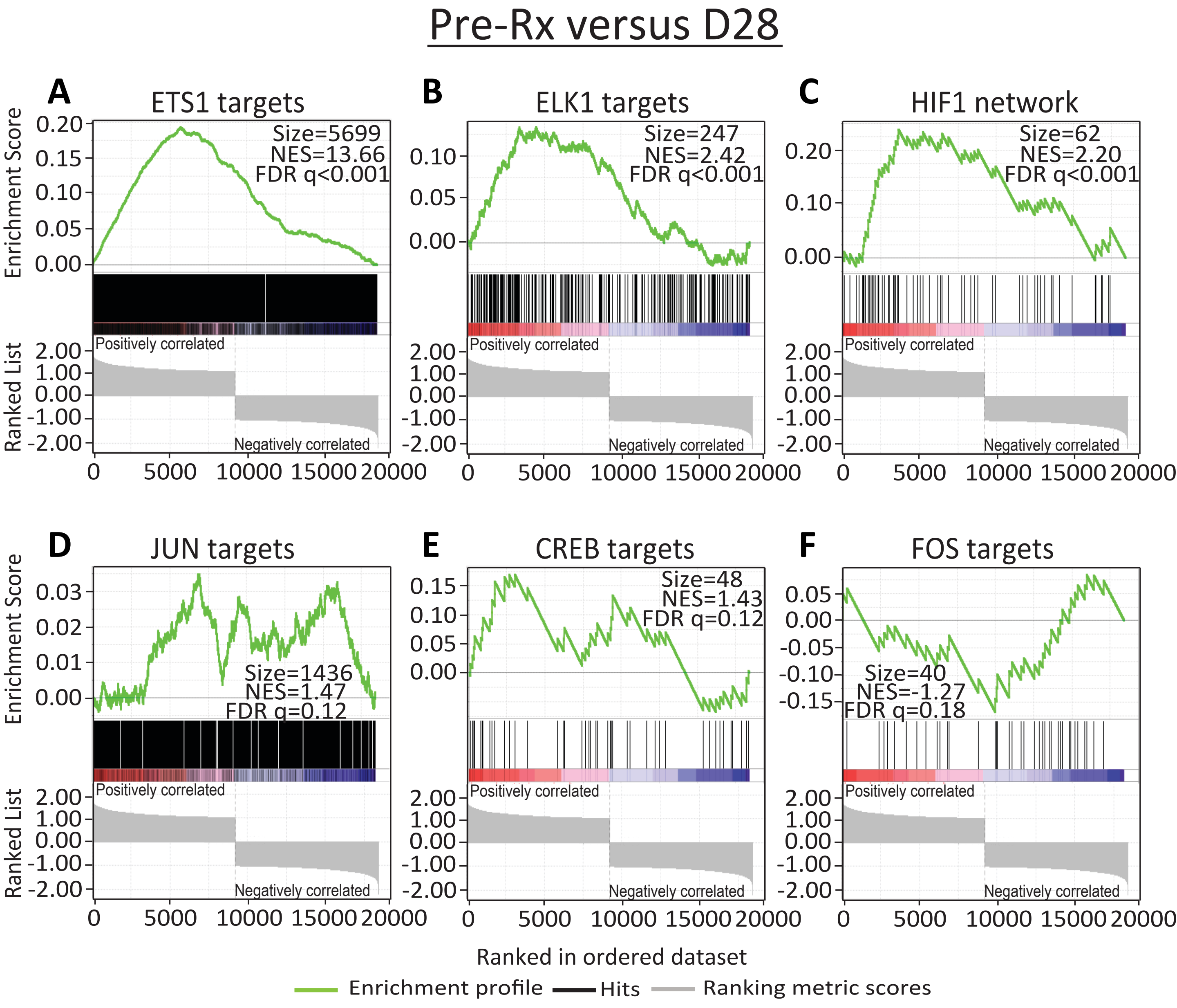

Supplementary Figure S2

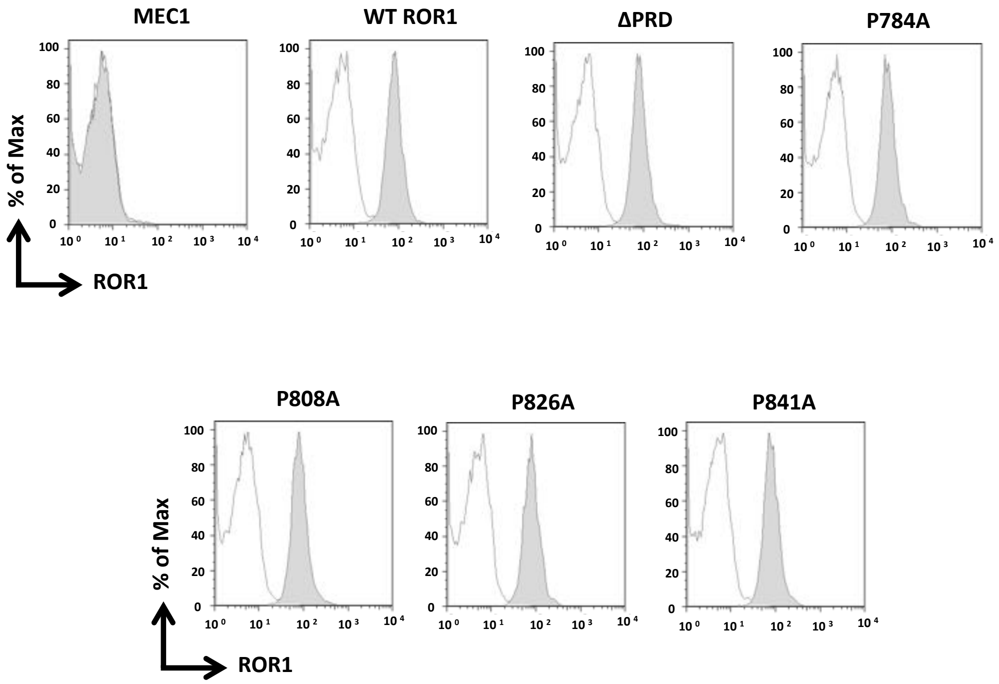

Supplementary Figure S3

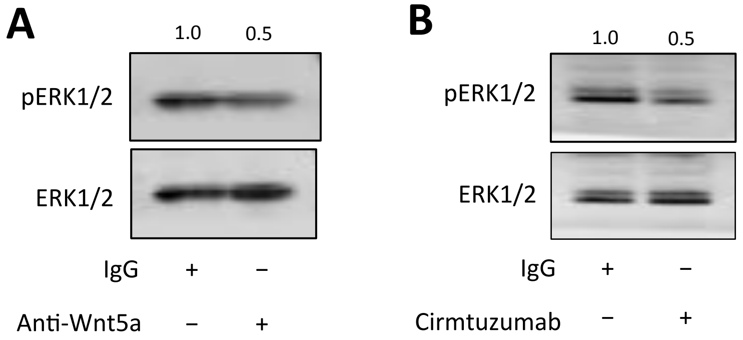

Supplementary Figure S4

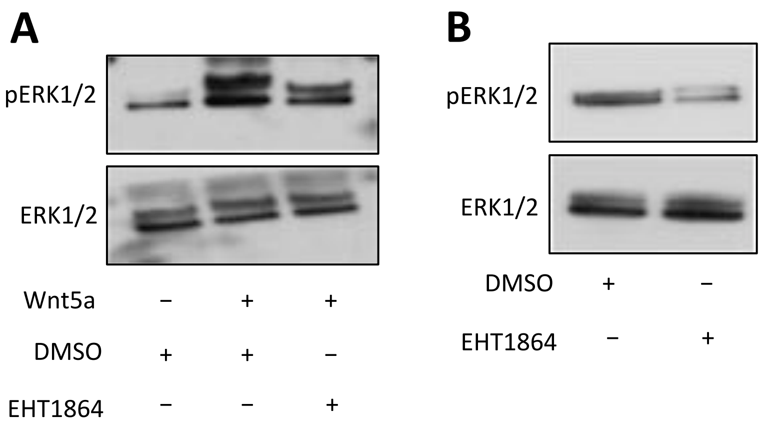

Supplementary Figure S5

DOCK2 tyrosine phosphorylation: DLIGKNVY(985)PGDWMAMSMVQNR

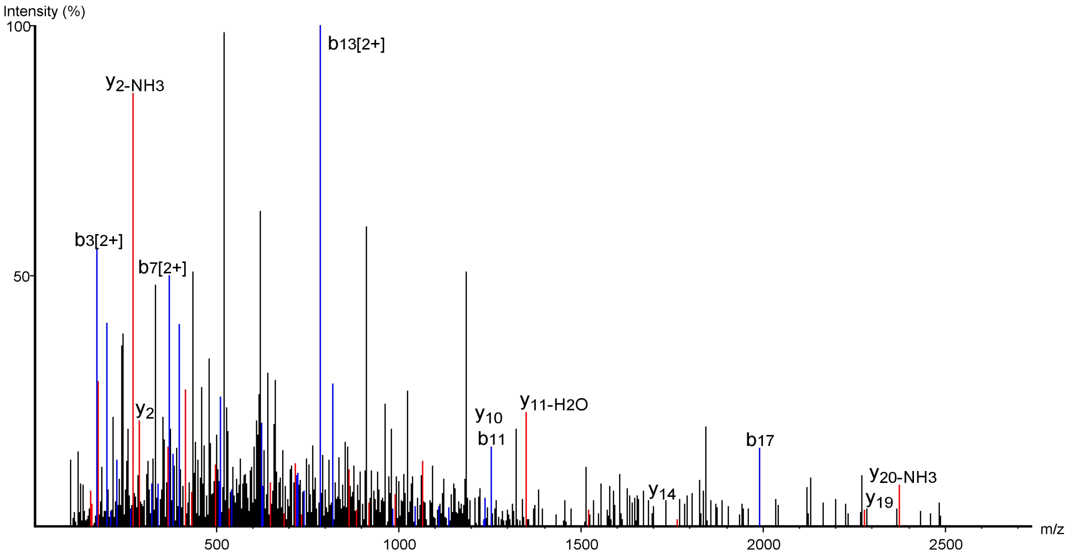

Supplementary Figure S6

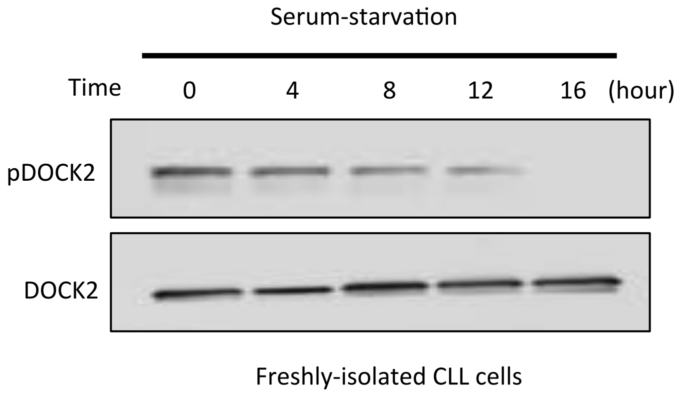

Supplementary Figure S7

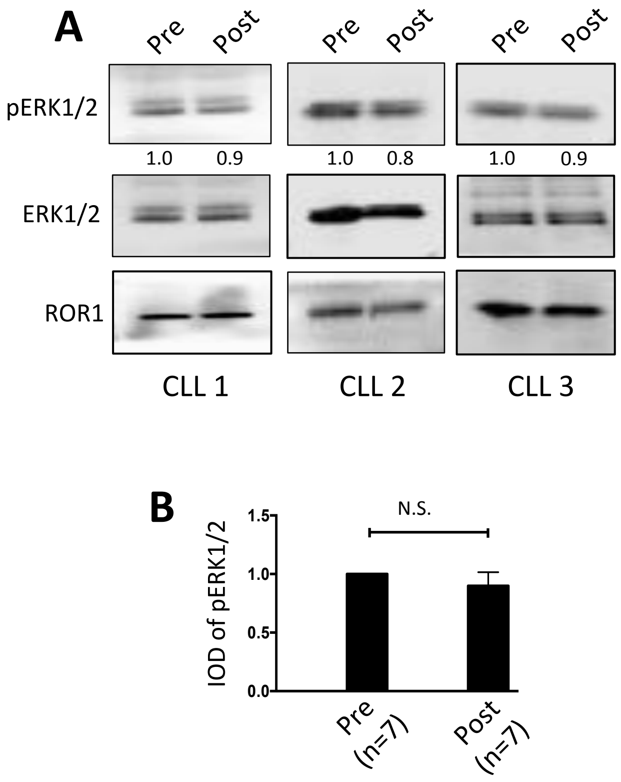

Supplement: Supplementary file 2 — Supplemental Figures [file 41375_2020_1055_MOESM2_ESM.pdf]
